# Tissue-resident immunity in the female and male reproductive tract

**DOI:** 10.1007/s00281-022-00934-8

**Published:** 2022-04-29

**Authors:** Dennis Yüzen, Petra Clara Arck, Kristin Thiele

**Affiliations:** Division of Experimental Feto-Maternal Medicine, Department of Obstetrics and Fetal Medicine, University Medical Center Hamburg-Eppendorf, Martinistr. 52 – 20246, Hamburg, Germany

**Keywords:** Cancer, Decidua, Endometrium, FRT, Immune memory, Pregnancy, Testis, Tissue-resident, Uterus, Vagina

## Abstract

The conception of how the immune system is organized has been significantly challenged over the last years. It became evident that not all lymphocytes are mobile and recirculate through secondary lymphoid organs. Instead, subsets of immune cells continuously reside in tissues until being reactivated, e.g., by a recurring pathogen or other stimuli. Consequently, the concept of tissue-resident immunity has emerged, and substantial evidence is now available to support its pivotal function in maintaining tissue homeostasis, sensing challenges and providing antimicrobial protection. Surprisingly, insights on tissue-resident immunity in the barrier tissues of the female reproductive tract are sparse and only slowly emerging. The need for protection from vaginal and amniotic infections, the uniqueness of periodic tissue shedding and renewal of the endometrial barrier tissue, and the demand for a tailored decidual immune adaptation during pregnancy highlight that tissue-resident immunity may play a crucial role in distinct compartments of the female reproductive tract. This review accentuates the characteristics of tissue-resident immune cells in the vagina, endometrium, and the decidua during pregnancy and discusses their functional role in modulating the risk for infertility, pregnancy complications, infections, or cancer. We here also review data published to date on tissue-resident immunity in the male reproductive organs, which is still a largely uncharted territory.

## Concept of tissue-residency

In various non-lymphoid tissues—predominately at barrier sites such as the skin, lung or intestinal mucosa—distinct subsets of immune cells form a pool of tissue-resident lymphocytes where they are retained upon, e.g., pathogen clearance or antigen encounter. These cellular subsets include conventional CD4^+^ and CD8^+^ T cells, but also so-called innate T cells, such as γδ T cells, mucosa-associated invariant T (MAIT) cells, natural killer T (NKT) cells, and innate lymphoid cells (ILCs).

The classical understanding of peripheral T cell function has long been that circulating thymus-derived naïve T cells enter secondary lymphoid organs such as the spleen and lymph nodes. Here, the T cells may be activated upon contact with antigens presented by specific antigen-presenting cells (APC). If antigen contact does not occur, naïve T cells egress the lymphoid tissue through the lymph vessels into the blood to patrol to another secondary lymphatic organ. Once naïve T cells encounter an antigen via their respective major histocompatibility complex (MHC), activation, and rapid proliferation follows, and the T cells leave the lymphatic tissue and migrate to, e.g., the site of infection as effector T (T_EFF_) cells. After executing their specific effector function, most of the T_EFF_ cells undergo apoptosis while a small fraction of T cells returns to secondary lymphoid organs to form a reservoir of immunological memory, which can be efficiently reactivated if the specific antigen is re-encountered. However, T cell memory function is not only maintained in secondary lymphoid organs, but additionally executed locally by tissue-resident memory T cells (T_RM_).

This has sparked the concept of a whole-body immune system rather than an immune system located in primary and secondary lymphoid organs. Experimental evidence of tissue-residency was initially generated in parabiosis experiments. Hereby, two congenic mice are surgically conjoined by their circulatory system. Circulatory T cells are subsequently being exchanged between the mice until an equilibrium is achieved between both hosts, while a significant fraction of cells remain immobile and resides in specific organs [1]. The proof of concept that these immobile, tissue-resident cells are a functionally relevant, autonomous subpopulation was provided by Wakim et al. and Gebhardt et al*.*, who described an enhanced protection from subsequent infection with herpes simplex virus (HSV) in naïve mice which had received skin grafts. These grafts were taken from donor mice upon clearance of HSV infection [2, 3]. Additional explant experiments further highlighted tissue-residency in an organ-specific manner. Here, pathogen-specific T cells remained in the tissue graft (ganglia, intestine) and became reactivated during a pathogen rechallenge of the recipient [4].

Studies performed outside the context of pathogen reencounters advanced the understanding of T_RM_ cells. These studies challenged the concept that T_RM_ cells are terminally differentiated, immediate responders, since epigenetic analyses revealed a signature which is more similar to circulating memory T cell subsets than recently activated effector T cells [5, 6].

**Fig. 1:**
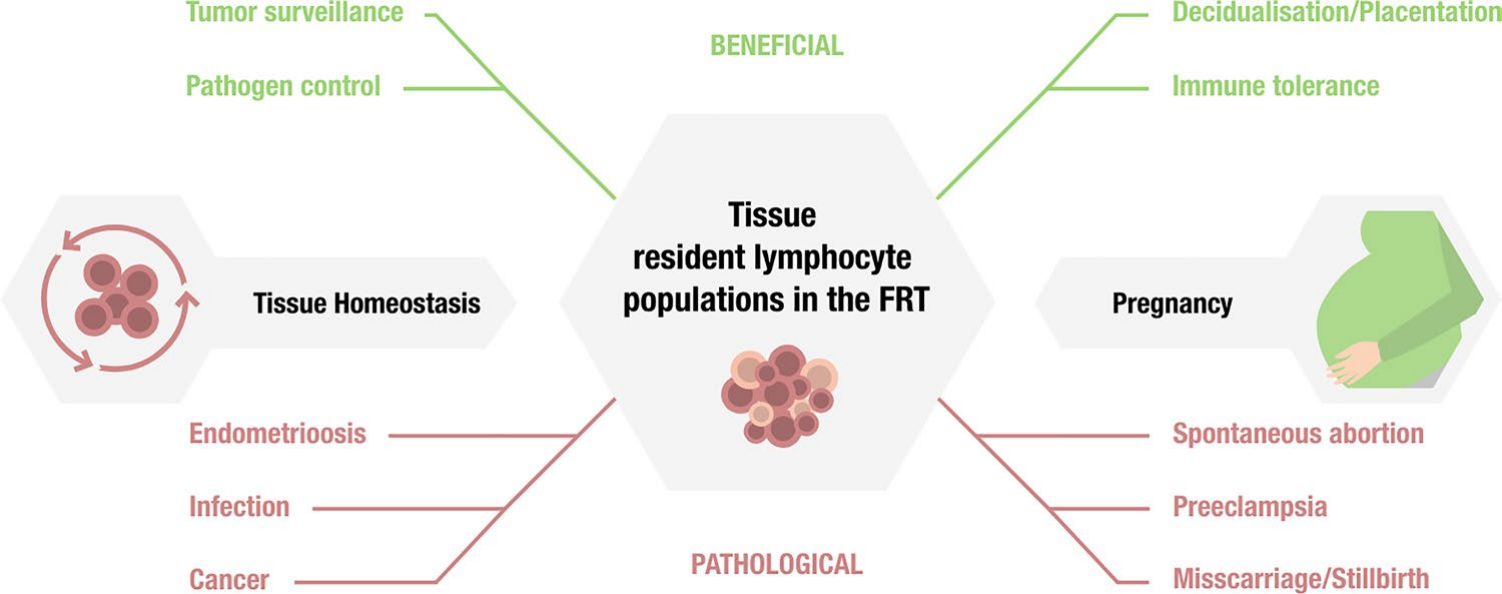
Functional diversity of tissue-resident lymphocytes in the female reproductive tract (FRT): Beneficial effects and pathological consequences in the context of tissue homeostasis and pregnancy

The functional role of T_RM_ cells can be subsumed as maintaining tissue integrity especially during infections, hereby restoring tissue homeostasis and protection from reinfection. Together with other tissue-resident lymphocyte populations that do not meet the classical definition of a memory cell in the context of infection, they are also engaged in tissue surveillance in malignancies, autoimmunity, and atopy [7–9]. However, tissue-resident immune cells also show a great degree of functional diversity, mirrored by beneficial as well as harmful effects for the host (Fig. 1). To date, tissue-resident immune cells have best been studied in epithelial barrier tissues in both, animal models and humans, including the gastrointestinal tract, lung and skin [10–13]. However, insights into the functional role of tissue-resident immunity in the female reproductive tract are surprisingly sparse.

## The Female Reproductive Tract

**Fig. 2:**
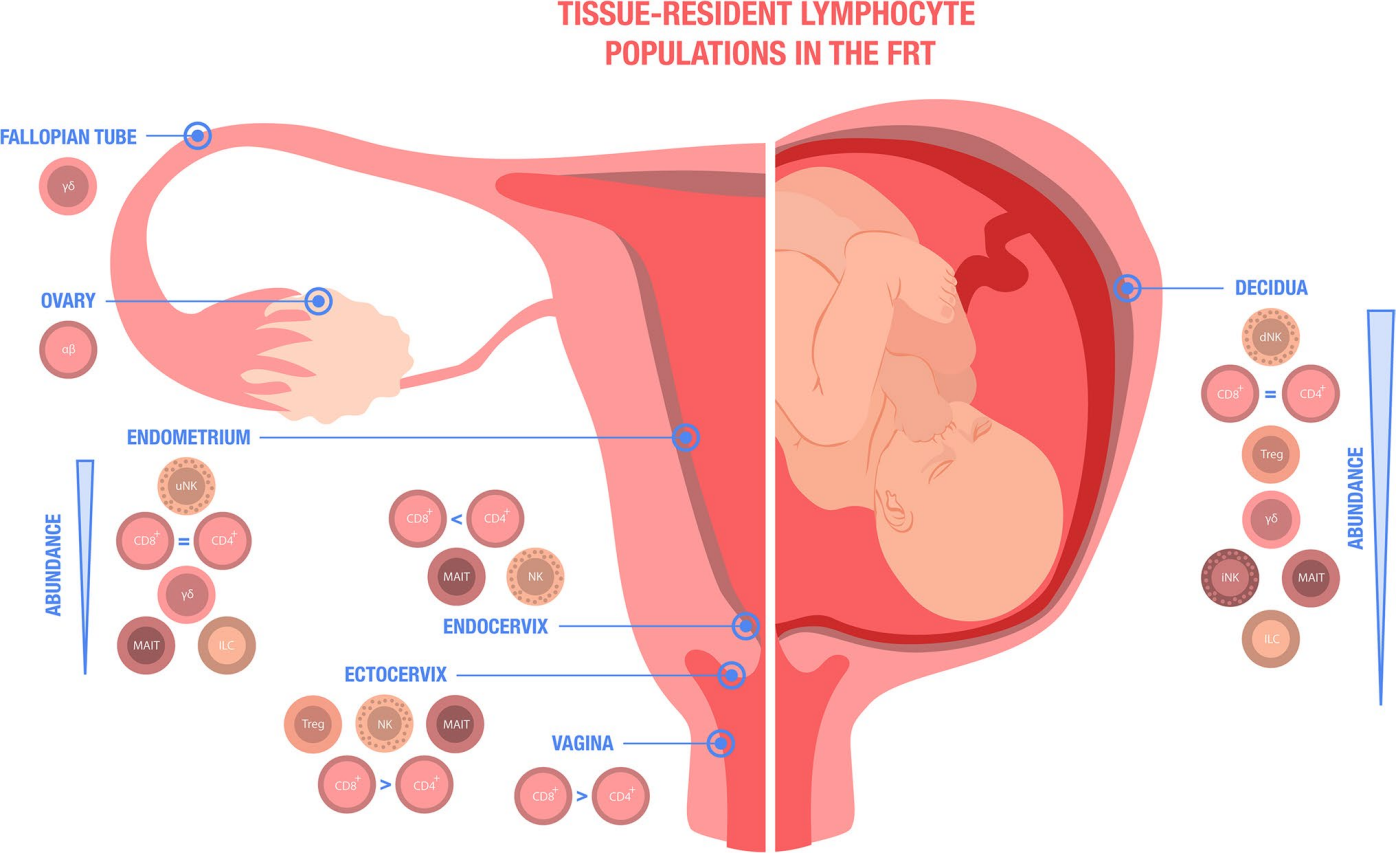
Female reproductive tract (FRT): Graphical summery of the presence of various tissue-resident lymphocyte populations including CD4^+^ and CD8^+^ T cells, CD4^+^ regulatory T (Treg) cells, γδT cells, mucosa-associated invariant T (MAIT) cells, uterine natural killer (uNK) and decidual natural killer (dNK) cells, invariant NKT (iNKT) cells, and innate lymphocyte cells (ILCs) in different compartments of the FRT in a non-pregnant state (left) and in the decidua during pregnancy (right)

Clearly, the female reproductive tract (FRT) shows a unique plasticity throughout life. Anatomically, it can be divided into two parts: the upper FRT is formed by the ovaries, the uterine tubes, the uterus, and the endocervix. The lower FRT consists of the ectocervix, the vagina, and the external genital organs [14]. As characteristic for barrier tissues, the FRT mainly consists of mucosal tissue that can be phenotypically and functionally divided into type I and type II. Type I mucosal surface consists of simple columnar epithelium while type II represents a stratified squamous epithelial layer. The ectocervix and the outer and inner vagina consist of type II mucosa, whereas the endocervix and the uterus is composed of type I mucosa [15]. The transition between type I and type II epithelium is referred to as cervical transformation zone [16]. In the following, we review the published evidence available to support the concept of tissue-resident immunity in the FRT. We hereby compartmentalize the FRT, as distinct anatomical regions can be anticipated to require a differential, site-specific tailored role of distinct subsets of tissue-resident immune cells (Fig. 2). An overview of phenotypical and functional characteristics of T_RM_ cells is provided in Table 1.

**Table 1:** Summary of T_RM_ subpopulation and the functional relevancy

Subset	Subtypes	Beneficial relevance	Pathological relevance	Reference
αβT cells	CD4^+^ CD8^+^	Pathogen clearance; Systemic activation of immune system; Differentiation to ex-T_RM_ cells; Contribute to local tumor surveillance	Infections	[4, 6, 47, 57, 136–138]
	CD4^+^ Treg cells CD8^+^ Treg cells	Mediate tolerance during pregnancy; Regulators in autoimmune diseases	Endometriosis; Pregnancy loss	
γδT cells	Vγ1-7 (Heilig and Tonegawa)	Contribute to local tumor surveillance; Immune homeostasis	RSA; Preterm birth	[90, 95, 139–141]
Mucosa-associated invariant T (MAIT) cells	MAIT1 MAIT2 MAIT17	Potential role in pathogen clearance	Preeclampsia	[98, 142–144]
Natural killer T (NKT) cells	Invariant NKT (iNKT) cells Variant NKT (vNKT) cells Non-classical NKT cells	Modulating the balance of Th1 and Th2 response; Contribute to local tumor surveillance	Preterm birth and fetal death; Preeclampsia	[53, 105, 107, 145]
Innate lymphocyte cells (ILCs)	Cytotoxic ILCs: NK cells	Mediate EVT invasion; Tumor surveillance	Infertility; RSA; Endometriosis	[53, 55, 146–151]
	Helper-ILCs: ILC1s ILC2s ILC3s Lymphoid tissue inducer (LTi)	Involved in pathogen clearance, tissue-repair and tumor surveillance	Placental abnormalities	

## Tissue-resident immunity in the vagina

The vaginal mucosa can be subjected to cohabitation and ejaculation of sperm, allowing sperm to enter the uterus through the cervix. An obvious need for tissue-resident immunity at the vaginal mucosa can be seen in the protection from sexually-transmitted diseases, such as chlamydia, gonorrhea, genital warts, syphilis, genital herpes, and human immunodeficiency virus (HIV). In fact, sexually transmitted diseases affect more than 300 million people every year and cause major health and pregnancy complications, such as an increased risk to acquire HIV, infertility, cancer, but also preterm or still birth. The understanding of vaginal tissue-resident immunity in the context of sexually-transmitted diseases is increasingly emerging, mostly from studies on HSV and HIV. Here, a well-conducted study highlights that memory CD4^+^ T cells provide protection from HSV-2 infection in mice [17]. These vaginal memory CD4^+^ T cells appear in clusters, which are maintained by local network of macrophage-derived chemokines and expanded in response to HSV-2 rechallenge. In human vaginal tissue, distinct subsets of APCs could be identified, which distinctly differ from other sites, such as the skin or gut mucosa [18]. There is evidence available to support that APCs may modulate the CD4^+^ and CD8^+^ T cell response in the vagina by inducing the expression of CD103 or chemokine receptors on T cells. Indeed, the majority of T cells have been identified as effector memory CD4^+^ T cells, co-expressing CD103 and the chemokine receptor 5 (CCR5). Contrary to the CD4^+^ T_RM_ cells studied in mouse vagina which reduced the risk for HSV, this human tissue-resident subset supported the infection with HIV-1. Interestingly, productive HIV-1 infection of these vaginal CD4^+^ T_RM_ cells was linked to the activation of uninfected bystander CD4^+^ T cells, which may amplify and facilitate the dissemination of the viral infection [19]. However, since disruption of the vaginal epithelium and related barrier breakage can aggravate HIV infection, tissue-resident CD4^+^ T cells may be more readily exposed to HIV-1, hereby triggering the infection. Clearly, further studies are urgently needed to identify the functional role of tissue-resident vaginal T cells in modulating the risk for sexually-transmitted diseases. Hereby, vaginal tissue-resident immunity must also be considered in post-menopausal tissues, considering that aging women are at increased risk for sexually-transmitted diseases [20].

Moreover, tissue-resident immunity in the vaginal mucosa can hold a great potential to maintain homeostasis and possibly protect from infections. In response to *Chlamydia muridarum* infection, a pathogen-specific subset of CD4^+^ T_RM_ cells is formed at the interface of the FRT epithelia and lamina propria, which mediates protection from secondary infection [21, 22]. Similarly, parenteral vaccination against *Chlamydia trachomatis* leads to the formation of a functional CD4^+^ T_RM_ cell subset in the genital tract with subsequent immunity [23]. Strikingly, locally applied vaccine strategies may establish protection from sexually transmitted diseases. It could be demonstrated that a combination of an intranasal and intravaginal mucosal immunization (“prime-boost immunization”) with recombinant influenza-HIV vectors results in a HIV-specific CD8^+^ T_RM_ population in the vaginal mucosa that led to the recruitment of peripheral adaptive and innate immune cells upon reactivation [24]. Another novel non-inflammatory vaccine strategy constitutes the “prime and pull” approach. After establishing a systemic memory response to HSV-2 infection in mice by conventional parenteral vaccination (prime), multiple topical chemokine applications onto the vaginal mucosa (pull) resulted in the infiltration of CD8^+^ T_RM_ cells and protection from reinfection [25]. In a follow-up study, the authors successfully demonstrated that a single topical application of the antibiotic neomycin onto the vaginal mucosa was sufficient to achieve a similar infiltration of virus-specific CD8^+^ T_RM_ cells with subsequent protection against genital HSV-2 infection [26, 27]. Despite concerns regarding collateral effects on the microbiome and artificial immune responses in contrast to recombinant chemokines, this prime and pull technique could be advantageous due to wildly availability, low-cost production and storage properties of Aminoglycoside antibiotics.

An alternative option to boost the formation of T_RM_ cells involves hormonal treatments. Hereby, the co-administration of estradiol after initial intranasal immunization with HSV-2 led to increased Th1 and Th17 T_RM_ cell frequencies with protective capabilities upon genital HSV-2 re-challenge [28].

However, the longevity of these protective T_RM_ cells is not fully understood. Evidence suggests that the T_RM_ compartment in the lower female reproductive tract is either short-lived—when compared to similar compartments in other barrier tissues—or tissue-resident cells may partly egress after a specific time period. Clearly, the latter would challenge their classification as tissue-resident cells [29].

An intriguing aspect in the context of tissue-resident immunity in the vagina is the impact of the microbiome. It is generally accepted that a microbiome-immunity crosstalk exists and contributes to various immune-mediated disorders [30]. Additionally, dysregulations of the microbiome in the genital tract could be linked to fertility [31] and obstetric complications such as miscarriage [32] and preterm labor [33–35]. Hence, tissue-resident immune cells may affect the diversity and composition of bacterial communities in the vagina, or vice versa, which may then become clinically evident [36].

Taken together, T_RM_ cells in the vagina play a major role in mediating resistance to viral and bacterial infections in the FRT. A stronger focus on the generation of functional tissue-resident memory subsets in vaccine development might be a promising addition to conventional vaccine approaches. Furthermore, although the presence of MAIT cells, invariant NKT cells, γδT-cells and ILC has been described in vaginal mucosal tissue [37, 38], their functional role, e.g., in responses to pathogens is relatively unknown. Hence, the analysis of these tissue-resident immune cell subsets should be considered in the experimental setup of future studies.

## Tissue-resident immunity in the endometrium

The endometrium lines the inner surface of the uterus and is structured in a basal and a functional layer. Immune cells can be found in the stromal compartment of both layers organized in lymphoid aggregates [39, 40]. Those aggregates consist of a B cell core surrounded by CD8^+^ T cells lined by macrophages [41]. The functional layer of the endometrial mucosa is subject of constant shedding and tissue-renewal due to the periodic remodeling during the menstrual cycle over the childbearing years. Initially, the menstrual cycle and monthly structural fluctuations seems to interfere with the concept of tissue residency. However, investigations of endometrial tissue during subsequent pregnancies revealed the expansion of CD8^+^ T_RM_ cells as well as ILC1 and NK cells suggesting a stable persistence in the basal layer during inter-pregnancy intervals [42–44]. Consequently, distinct endometrial tissue-resident lymphocytes contribute to tissue homeostasis and enable situational adaptations in the presence or absence of conception, but have also been linked to various immunopathologies and cancer.

The proliferative, secretory, and regenerative phases of the menstrual cycle affect the proliferative capacity of immune cells [45]. In cell culture experiments, the proliferation of peripheral blood mononuclear cells (PBMCs) is differentially inhibited by uterine cells isolated during the proliferative or the secretory phase of the menstrual cycle [46]. Under physiological conditions, CD4^+^ and CD8^+^ T cells are expressed at balance in the endometrium and by expressing CD103 and CD69 both comply with the canonical phenotype of T_RM_ cells [47–49]. However, during the secretory phase of the menstrual cycle the number of endometrial cytotoxic CD8^+^ T cells is decreased [50], which might hamper immune response toward the implanting conceptus. CD8^+^ T_RM_ cells in the endometrium that possess cytotoxic properties might be involved in secondary pathogen encounter [51]. Nevertheless, their exact function needs to be further elucidated.

Clearly, uterine NK (uNK) cells are the predominant lymphocyte population in the endometrium [52, 53]. Their most critical role seems to surface during early pregnancy, when they maintain tissue-homeostasis and promote angiogenesis. It has been suggested that uNK cells, especially tissue-resident uNK cells, play a role in endometriosis. Endometriosis is defined by the growth of endometrium-derived tissue outside the uterus. It chronically affects around 10% of women [40, 54], whereas its pathogenesis is far from being understood. Opposed to their phenotype in blood, uNK cells are uniquely defined by CD56^bright^ and CD16^neg^ expression, and tissue-resident uNK cells further express CD49a^+^. [55] Interestingly, in endometriosis patients, uNK cells exhibit an increased cytotoxic phenotype, mirrored by an elevated CD16^+^ and NKp46 expression [56]. Together with an increased endometrial cell expression of MHC Class I molecules, this could favor the migration of abnormal ectopic endometrium. Additionally, peripheral and peritoneal NK cells show increased expression of inhibitory killer cell immunoglobulin-like receptor (KIRs), which might further contribute to a reduced removal of endometrial cells by NK cells outside the uterus. Further, lower CD4^+^ regulatory T (Treg) cells and greater T helper (Th)17 cell frequencies in the endometrial tissue favors local inflammation in ectopic and endometrial tissues [57, 58]. On the contrary, there is evidence to support that ectopic endometrial tissue harbors CD4^+^ Treg cells, which reduced recognition and rejection of ectopic endometrial by effector immune cells, e.g., in the peritoneal cavity [58].

Interestingly, endometriosis often occurs together with infertility; the overlap ranges from 40–50% [59]. Infertility affects millions of people worldwide and is defined by the failure to successfully achieve pregnancy after more than 12 months of regular unprotected sexual intercourse.^1^ In women with endometriosis-associated infertility, low levels of endometrial stem cell factor has been observed, which suggests that the maturation of local uNK cell populations is impaired, which subsequently compromises embryo implantation [60]. This notion is supported by the observation that a higher number of uterine CD34^+^ NK cell progenitors in women with endometriosis is positively correlated with sustained fertility [61].

Tissue-resident immunity in the FRT may affect tumor-surveillance and control of cancer progression. This research field is of particular importance since three of eight cancer types with the highest incidence in women emerge in the FRT including cervical cancer (13,3%), uterine cancer (8,7%) and ovarian cancer (6,6%), which constitute a threat to women’s health and survival.^2^ Since cancer is at least partly a result of T cell dysregulation, insights into T_RM_ cells located in the FRT are highly relevant for understanding cancer development and illustrating treatment methods [62]. Recently, it became evident, that γδT cells are especially involved in tumor-surveillance. γδT cells colonize the FRT in mice already during fetal and neonatal development. Although uterine location is no further specified, γδT cells are the dominant T cell subpopulation in the uterus of neonatal mice accounting for more than 50%. Interestingly, their number declines with advancing age, resulting in less than 20% of overall T cells at 16 weeks of age [63]. Uterine γδT cells are located primarily in the intraepithelial compartment but recent data suggest an alternative location in the subepithelial stroma of the uterus [63]. The majority of γδT cells in the endometrium belongs to the Vγ6^+^ subgroup producing IL-17 upon activation, but a discrete population of IFNγ-producing γδT cells was observed in the murine uterus [64]. Dependent on the cytokine specificity, γδT cells exhibit functionally diverse responses to tumors and the microenvironment. Anti-tumor effects are characterized by cytotoxicity against hematopoietic and solid tumors in an MHC-independent manner [65]. Both Vδ2 and Vδ1 subsets produce IFNγ, which may induce the elimination of carcinoma cells. In contrast to the potent anti-tumor capacity, γδT cells are also able to induce pro-tumor effects, facilitating non-cytotoxic inflammation and angiogenesis via IL-17 production. Additionally, a subset of γδT cells is suggested to exert regulatory functions, these are referred to as γδTreg cells. In breast cancer, these cells were shown to contribute to an immunosuppressive microenvironment and induce the immunosenescence of T_EFF_ cells and dendritic cells (DCs) [66]. The functional diversity of γδT cells in the context of tumor development in the FRT needs further investigation to advance the potential of immunotherapy in cancer treatment strategies.

Besides γδT cells, tumor infiltrating lymphocytes (TILs) expressing the surface makers CD8^+^ and CD103^+^ are present in tumor tissue and classified as T_RM_ cells [67, 68]. They are associated with a prolonged survival prognosis in cervical, endometrial, and ovarian cancer [69–72]. Tissue-resident TILs often express the T cell exhaustion marker Programmed cell death protein (PD)-1, which get activated by its ligand PD-L1 on cancer cells [73] switching TILs into a dormant state. Hence, recent strategies in tumor treatment pursue the application of checkpoint inhibitors, blocking either PD-1 or PD-L1 in order to reactivate TILs [74].

Another promising approach of treatment option represents a NK cell-based anti-cancer immunotherapy, which is exploiting the potential of NK cell to infiltrate tumor tissue and to kill malignant cells. Based on high NK cell frequencies and high prevalence of tumor formation in the FRT, strategies for increasing tumor recognition by NK cells have been discussed. This includes the sensitizing of tumor cells for NK cell killing, improving the cytotoxicity of NK cells ex-vivo via cytokine treatment or the generation of tumor-specific NK cells generated via genetic engineering using chimeric antigen receptor (CAR)-expressing NK cells [75, 76].

Further, a major advantage of T_RM_ cells is their location in the periphery of the body. In the context of cancer treatment, this ability could be utilized by making T_RM_ cells a vigilant ally in fighting metastasis in an early disease state. Accordingly, T_RM_ cells might be a promising target for future cancer treatment not only in the FRT.

A potential role of tissue-resident immunity in the endometrium is also discussed in the context of sexually transmitted diseases. Recent evidence supports the capability of MAIT cells to respond to *N. gonorrhoeae* infection with a specific cytokine response, as shown in during in-vitro experiments [77]. However, solid studies on MAIT cell function in the context of protection against infections in the FRT are still lacking, although they form a stable population in the endometrium and the cervix which is unaffected by phases of the menstrual cycle as well as during menopause [77].

For the sake of completeness, it needs to be mentioned that only very few information is available concerning T_RM_ populations in the ovary and fallopian tube. Only few leukocytes are located in the human ovary, but immunohistochemistry and single-cell analysis identified CD45RO^+^ and CD69^+^ T cells, suggesting a viable T_RM_-compartment [78, 79]. In the fallopian tube, γδT cells can be found located in the epithelial layer and lamina propria of the mucosa [80].

## Tissue-residency in the decidua during pregnancy

Significant adaptions of the endometrium occur with the onset of pregnancy. The endometrium undergoes decidualization, a process tightly regulated by hormonal changes. Additionally, placentation in mammals involves the deep invasion of extra-embryonic placental cells into the maternal decidua [81]. This invasion results in close contact between fetal trophoblasts, which express paternally-inherited foreign antigens, and maternal immune cell populations. This requires a unique immune regulation in order to prevent fetal rejection.

One key element of fetal acceptance is the lack of MHC class 1 (except HLA-C) and MHC class II receptors on human EVTs, but the presence of all three non-classical MHC class I antigens (HLA-E, HLA-F, and HLA-G) [82]. Hence, an anti-fetal maternal immune response is diminished, but the recognition of the fetal antigen is not fully disabled. Consequently, a tailored maternal immune response needs to be initiated in order to mount immune tolerance toward the allogeneic fetal trophoblast cells. This includes the arrest of DCs in a tolerogenic state and subsequently the priming and expanding of CD4^+^ Treg cells [7]. A decisive impact of T_RM_ cell subpopulation on this active immune adaptation at the feto-maternal interface is indicated, although still under intensive investigation. Therefore, adverse pregnancy outcomes such as recurrent spontaneous abortion (RSA), miscarriage, stillbirth, preeclampsia, and preterm birth can be partially linked to immune dysregulation of tissue-residency in the FRT.

In this context, decidual NK (dNK) cells may have the most critical function. They represent up to 70% of decidual lymphocytes in human first trimester pregnancy and 30% in murine decidua at midgestation [83, 84]. The ontogeny of dNK cells is still subject of ongoing debate. It is possible that NK cells in the peripheral blood migrate to the decidua, attracted by the unique decidual microenvironment [85]. However, CD34^+^ precursor cells are detectable in the decidua and immature uNK cells can be found in the endometrium of non-pregnant women, which could be evidence for in situ generation of NK cells [86]. In mice and humans, dNK recognize HLA/MHC on trophoblast cells, e.g., in humans via the C-type lectin-like CD94/NK group 2 (NKG2) receptors and KIR [83]. HLA-C/KIR-mismatches have a high predictive value for poor placentation and impaired continuation of pregnancy. In this regard, although still conflicting, the genetic variability of maternal KIR paired with fetal HLA-C have been associated with the pathophysiology of preeclampsia suggesting that inhibitory KIR (KIR AA genotype) negatively impact uNK cytokine secretion leading to abnormal spiral artery remodeling and defective placentation [87]. Thus, selecting suitable HLA-C/KIR-matches by screening for HLA-C subtypes could be a promising tool to increase the success rate of modern assisted reproduction technologies [88].

It was further proposed, that dNK cells might support implantation and placentation in subsequent pregnancies by acquiring a memory-like phenotype during the first pregnancy [44]. Although these “pregnancy-trained” dNK cells were shown to exhibit a unique transcriptional and epigenetic phenotype, their abundance could only be confirmed in Cytomegalovirus (CMV)-positive pregnant women [44, 89]. Hence, further studies are required to clarify if and how CMV might facilitate the formation of trained memory dNK cells in contrast to a more generalized beneficial effect for multigravidity.

Besides dNK cells, γδT cells are also present in the decidua. These decidual γδT cells execute important functions ensuring local immune homeostasis by shaping pro- and anti-inflammatory responses. They are part of the decidua-associated lymphoid tissue (DALT), which comprise of approx. 15% of the decidual T cell pool [90, 91]. In contrast to Vδ2^+^ γδT cells, which are dominant in blood, the vast majority of human decidual γδT cells are Vδ1^+^. This subset actively promotes trophoblast invasion in the maternal decidua and suppresses trophoblast apoptosis. This is mediated by IL-10 secretion of γδT cells, accompanied by reduced granzyme B secretion following chemokine–receptor interaction with trophoblast cells [90, 92]. After initiation of pregnancy, the composition of γδT cell subsets fluctuate according to progesterone levels [93, 94]. During the second trimester of pregnancy, the ratio of Vδ2^+^ to Vδ1^+^ γδT cells is increasing. A premature increased Vδ2^+^/Vδ1^+^ γδT ratio in the first trimester of pregnancy is linked to spontaneous abortion due to a premature proinflammatory environment [90]. Hereby, γδT cells modulate the Th1/Th2 ratio observed by an increased Vδ2^+^ γδT cell count leading to an increase of Th1 cells at the decidua. Th1 cells act in a proinflammatory manner compared to their counterparts reversing the immune tolerant state at the feto-maternal interface and therefore steadily contribute to onset of childbirth. Therefore, an association of altered γδT frequencies and preterm birth could be conceivable [95]. In the term decidua, the majority of γδT cells belongs to the naive/memory and translational phenotype [94]. Taken together, γδT cell function at the feto-maternal interface is highly flexible and depends on the state of pregnancy, although additional investigations are indispensable to further ascertain the impact of γδT cells during pregnancy.

A sizable threat for pregnancy success constitutes microbial infection. Hereby, MAIT cells located in the intravillous space of the placenta express higher levels of IFNγ and granzyme B upon microbial stimulation compared with their circulatory counterparts [96]. MAIT cells are enriched in the placenta and the decidua. They remain relatively stable over the course of pregnancy [97], but exhibit a distinct phenotype compared to MAIT cells in the blood or the endometrium. At term, MAIT cells accumulate within the intervillous space of placenta displaying an increased inflammatory response to riboflavin-producing bacteria [97]. The specific functionality of MAIT cells in maintaining a healthy pregnancy is still a matter of investigation. Hereby, the interaction of MAIT cells with EVTs remains particularly uncertain since MAIT cells are not able to recognize HLA-molecules on the EVT surface. In contrast to fetal macrophages located in fetal villi, the syncytiotrophoblast does not express monomorphic MHC-like receptor 1 (MR1) molecule also contradicting an antagonistic interaction between MAIT cells and EVTs. Nevertheless, a recent study observed an altered frequency and reduced PD-1 expression of MAIT cells in PBMCs of women with early-onset-preeclampsia [98]. Hence, an in-depth investigation of the local uterine MAIT cell population might contribute to an improved understanding of the pathogenesis of preeclampsia.

Regulating immune homeostasis during pregnancy is further supported by decidua-invariant natural killer T (iNKT) cells showing a tenfold increase in number compared to peripheral blood [99]. Evidence suggests that iNKT cells interact with extravillous and villous trophoblast cells both expressing CD1d [100]. The expression level of CD1d even increases with progressing gestation [101]. However, a recent publication questioned the importance of iNKT cell in pregnancy since less than 1% of the CD56^+^ CD3^+^ NKT cells are also positive for the iNKT-specific CD1d tetramer [102]. Despite discussion regarding their actual proportion at the feto-maternal interface, a shift toward a Th1-biased cytokine profile of iNKT cells, including increased TNFα, IFNγ and perforin production, seems to contribute to higher pregnancy loss rates [103, 104]. Further, upon iNKT cell activation also local dNK cells start producing IFNγ supporting NKT cell-mediated pregnancy loss [101]. As a proof of concept, fetal death rates, but also preterm birth, could be reduced in iNKT cell-deficient mice after LPS challenge [105, 106]. Due to their ability to modulate Th1/Th2-balance, an iNKT-dysregulation is also assumed in preeclampsia. It was shown that preeclamptic women display elevated levels of Th1-type cells as a result of iNKT malfunction [107].

Emerging evidence further suggests a critical role for helper ILCs in promoting immune responses at barrier surfaces including inflammatory and reparative responses [108]. All ILC subtypes were shown to be present in the human and mouse uterus. In the human decidua IFNγ-producing ILC1 and subpopulations of ILC3 were identified in the first trimester of pregnancy [109]. ILC3s were observed to express PD-1 interacting with PD-1L^+^ trophoblasts to induce a tolerant microenvironment [110]. However, toward the end of pregnancy, ILC2s become the prevalent subtype of ILCs [111]. They get activated by thymic stromal lymphopoietin which was independently shown to be crucial for normal pregnancy by promoting the invasion of human trophoblasts and interacting with DCs and CD4^+^ Treg cells [112]. Interestingly, ILC were shown to contribute to an effective recall response upon reactivation. However, in contrast to adaptive immune cells, ILCs get reactivated by cytokines and therefore this effect is not antigen specific [113]. In the context of pregnancy, memory capacity was only reported for ILC1 showing an 4–fivefold increase in frequency in a second pregnancy uterus with upregulation of the memory cell marker CXCR6 [43].

The simultaneous upregulation of exhaustion-related molecules such as PD-1 to induce a tolerant phenotype is also reported for CD8^+^ CD69^+^ CD103^+^ T_RM_ cells [114, 115]. Those changes are mediated by decidual stromal cells facilitating the silencing of cytotoxic immune cells accompanied by CD4^+^ Tregs classifying the uterine mucosa as an immunologically privileged site [116, 117]. After successful completion of pregnancy, the composition of the endometrium must be restored to enable the periodic remodeling of the menstrual cycle again and subsequent re-conception which might be facilitated by T_RM_ cells. They could contribute to the reduced risk of complications during second pregnancies if regulatory T_RM_ cells generated during a first pregnancy become rapidly available [118]. However, research evidence regarding the presence of regulatory T_RM_ cells in the uterus are sparse and consequently their contribution to repeated pregnancy success requires further investigation.

## Uncharted territory: The male reproductive tract (MRT)

The MRT consists of external and internal organs including the penis and scrotum and the testis, epididymis, vas deferens and the accessory glands, respectively. Similar to females, the MRT is mainly lined by mucosal tissue [14]. However, compared to the FRT, the MRT is not a classical barrier tissue due to its limited exposure to the environment. Hence, the MRT is not as susceptible to infections due to the smaller surface area exposed to pathogens and the shorter contact time with pathogens before clearance [119]. Nevertheless, MRT infections and sexually transmitted diseases are also a major health burden in men underlining the importance of a responsive local immune environment.^3^ Hence, it is even more surprising how little is known about tissue-resident immune cell subsets in the MRT.

The primary infection site of the MRT is the penile urethra and CD103^+^ CD8^+^ T_RM_ cells were shown to be involved in microbial immune surveillance [120]. However, infectious agents are able to ascend to the testis, which can be especially harmful. The testis displays a unique anatomy. In order to prevent immune activation by sperm autoantigens, the seminiferous epithelium, the site of spermatogenesis, is separated from the interstitium by the blood testis barrier (BTB), leaving the seminiferous tubules an immunoprivileged site [121]. That comprises the total absence of lymphocytes in the seminiferous epithelium. Although being beneficial for spermatogenesis, this leads to the testis being an applicable reservoir for infectious agents after acute infection including HIV, human papillomavirus (HPV) or *Chlamydia trachomatis* leading to chronic infections [122]. This highlights the conflict between guarantee of self-tolerance by the absence of a viable immune cell compartment on the one hand and being susceptible to infections on the other hand.

In contrast to the seminiferous epithelium, distinct T_RM_ cells are present within the interstitial space of the testis [123–126]. The proliferation of especially CD4^+^ Treg cells is facilitated by the secretion of immune modulatory factors of sertoli cells and myeloid cells [127]. This is further promoted by the egress of autoantigens passing the BTB, educating local immune cells and therefore contributing to tissue homeostasis [128]. Infections or cancerous diseases are able to challenge this delicate balance leading to autoimmune orchitis, which is characterized by testis inflammation and the presence of specific antisperm antibodies and can result in aspermatogenesis and male infertility [129].

In a model of experimental autoimmune orchitis (EAO), the number of T cells were shown to be increased and their exact composition varied during disease progression [8]. In subsequent experiments, it could be further demonstrated that CD4^+^ Treg cells in allografts originating from rats suffering from EAO inhibit the proliferation of effector T cells in healthy animals [8]. This elegantly highlights the need of regulatory lymphocytes in chronic inflammatory diseases to confine an overshooting effector immune response. Despite the possibility that these CD4^+^ Treg cells could migrate from the surrounding lymph nodes into the testicular tissue during disease progression, it is highly probabilistic that a stable compartment of regulatory T_RM_ cells are permanently present within the tissue.

This assumption is supported by the ontogeny of the γδT cell compartment in the MRT. There is evidence that a viable γδT cell subset is present in the human semen [130]. Subsequently, γδT cells were also found in the rodent testis, already colonized during fetal development, where they strongly expand during puberty and form a tissue-resident subset residing until adulthood [131]. In contrast to CD4^+^ Treg cells, γδT cells are in fact involved in maintaining tissue-homeostasis. As demonstrated in *in-vitro* experiments with *Listeria monocytogenes,* γδT cells operate as immune-regulatory mediators following infectious stimuli. [125, 132].

The role of T_RM_ cells in tumor surveillance is barely investigated and this lack of scientific data is highly problematic since testicular cancer is the most commonly diagnosed malignancy in younger men [133]. A germ cell tumor is diagnosed in 95% of these cases making it the most prevalent cancer type in the MRT [134]. Interestingly, testicular cancer comprises only 1% of all male cancers globally, which is surprisingly low considering that the testis is a site of immune privilege. Hence, it may constitute a prominent site for tumor growth which is supported by the testis serving as a reservoir of relapse cancers, e.g., acute lymphocytic leukemia (ALL) [135]. Since reliable data regarding pro- and anti-tumor responses in the MRT are missing, we can only assume that the underlying mechanisms might be similar as observed in the FRT or other tissues.

In summary, our general understanding of tissue-resident immunity in the MRT is still fragmentary. However, the increasing relevance of T_RM_ cells throughout the body will contribute to new insights on the MRT and their functional impact for local immune homeostasis.

## Concluding remarks

The identification of T_RM_ cells has fundamentally changed our understanding of adaptive immunity and immunological memory especially at barrier sites. Their resident phenotype represents a major advantage leaving T_RM_ cells in a superior position to provide immediate protection to secondary infection hereby preventing dissemination of pathogens. However, T_RM_ cells display a significant functional diversity. While supporting tissue homeostasis including tumor surveillance and pathogen control, T_RM_ cells can also contribute to tumor growth and infections. In the context of reproduction, T_RM_ cells facilitate decidualization and placentation hereby supporting pregnancy establishment and maintenance, but also participate in the pathogenesis of various obstetric complications. In contrast to other barrier sites, the FRT needs to sustain a unique plasticity throughout life to ensure proper function during different phases of life and reproductive demands hereby preventing infections and cancer development. Hence, a comprehensive investigation of tissue-resident immunity in the FRT is urgently needed to advance our understanding of T_RM_ cell composition, phenotype and activation, and hormonal responsiveness and consequently of pregnancy success and failure.

## Footnotes

1 World Health Organization (WHO). International Classification of Diseases, 11th Revision (ICD-11) Geneva: WHO 2018.

2 Global Cancer Observatory (GCO), World Health Organiaztion. Last accessed 31.01.2022: https://gco.iarc.fr

3 Sexually Transmitted Diseases Surveillance 2019, CDC. Last accessed 31.01.2022: https://www.cdc.gov/std/statistics/2019

## References

[1] Klonowski KD, Williams KJ, Marzo AL, Blair DA, Lingenheld EA, Lefrancois L (2004). Dynamics of Blood-Borne CD8 Memory T Cell Migration In Vivo. Immunity.

[2] Gebhardt T, Wakim LM, Eidsmo L, Reading PC, Heath WR, Carbone FR (2009). Memory T cells in nonlymphoid tissue that provide enhanced local immunity during infection with herpes simplex virus. Nat Immunol.

[3] Wakim LM, Waithman J, van Rooijen N, Heath WR, Carbone FR (2008). Dendritic Cell-Induced Memory T Cell Activation in Nonlymphoid Tissues. Science.

[4] Wijeyesinghe S, Beura LK, Pierson MJ, Stolley JM, Adam OA, Ruscher R, Steinert EM, Rosato PC, Vezys V, Masopust D (2021). Expansible residence decentralizes immune homeostasis. Nature.

[5] Fonseca R, Beura LK, Quarnstrom CF, Ghoneim HE, Fan Y, Zebley CC, Scott MC, Fares-Frederickson NJ, Wijeyesinghe S, Thompson EA, Borges da Silva H, Vezys V, Youngblood B, Masopust D (2020). Developmental plasticity allows outside-in immune responses by resident memory T cells. Nat Immunol.

[6] Klicznik MM, Morawski PA, Hollbacher B, Varkhande SR, Motley SJ, Kuri-Cervantes L, Goodwin E, Rosenblum MD, Long SA, Brachtl G, Duhen T, Betts MR, Campbell DJ, Gratz IK (2019). Human CD4(+)CD103(+) cutaneous resident memory T cells are found in the circulation of healthy individuals. Sci Immunol.

[7] Jorgensen N, Persson G, Hviid TVF (2019). The Tolerogenic Function of Regulatory T Cells in Pregnancy and Cancer. Front Immunol.

[8] Jacobo P, Guazzone VA, Jarazo-Dietrich S, Theas MS, Lustig L (2009). Differential changes in CD4+ and CD8+ effector and regulatory T lymphocyte subsets in the testis of rats undergoing autoimmune orchitis. J Reprod Immunol.

[9] Webb JR, Milne K, Watson P, deLeeuw RJ, Nelson BH (2014). Tumor-Infiltrating Lymphocytes Expressing the Tissue Resident Memory Marker CD103 Are Associated with Increased Survival in High-Grade Serous Ovarian Cancer. Clin Cancer Res.

[10] FitzPatrick MEB, Provine NM, Garner LC, Powell K, Amini A, Irwin SL, Ferry H, Ambrose T, Friend P, Vrakas G, Reddy S, Soilleux E, Klenerman P, Allan PJ (2021). Human intestinal tissue-resident memory T cells comprise transcriptionally and functionally distinct subsets. Cell reports.

[11] Clarke J, Panwar B, Madrigal A, Singh D, Gujar R, Wood O, Chee SJ, Eschweiler S, King EV, Awad AS, Hanley CJ, McCann KJ, Bhattacharyya S, Woo E, Alzetani A, Seumois G, Thomas GJ, Ganesan A-P, Friedmann PS, Sanchez-Elsner T, Ay F, Ottensmeier CH, Vijayanand P (2019). Single-cell transcriptomic analysis of tissue-resident memory T cells in human lung cancer. J Exp Med.

[12] Son YM, Cheon IS, Wu Y, Li C, Wang Z, Gao X, Chen Y, Takahashi Y, Fu Y-X, Dent AL, Kaplan MH, Taylor JJ, Cui W, Sun J (2021). Tissue-resident CD4(+) T helper cells assist the development of protective respiratory B and CD8(+) T cell memory responses. Science immunology.

[13] Park SL, Buzzai A, Rautela J, Hor JL, Hochheiser K, Effern M, McBain N, Wagner T, Edwards J, McConville R, Wilmott JS, Scolyer RA, Tüting T, Palendira U, Gyorki D, Mueller SN, Huntington ND, Bedoui S, Hölzel M, Mackay LK, Waithman J, Gebhardt T (2019). Tissue-resident memory CD8+ T cells promote melanoma–immune equilibrium in skin. Nature.

[14] Sulaiman S, Coey J, Carrol M (2018). Male and Female Reproductive Anatomy. Clinical Reproductive Science.

[15] Iwasaki A (2010). Antiviral immune responses in the genital tract: clues for vaccines. Nat Rev Immunol.

[16] Kumamoto Y, Iwasaki A (2012). Unique features of antiviral immune system of the vaginal mucosa. Curr Opin Immunol.

[17] Iijima N, Iwasaki A (2014). T cell memory. A local macrophage chemokine network sustains protective tissue-resident memory CD4 T cells. Science.

[18] Duluc D, Gannevat J, Anguiano E, Zurawski S, Carley M, Boreham M, Stecher J, Dullaers M, Banchereau J, Oh S (2013). Functional diversity of human vaginal APC subsets in directing T-cell responses. Mucosal Immunol.

[19] Saba E, Grivel JC, Vanpouille C, Brichacek B, Fitzgerald W, Margolis L, Lisco A (2010). HIV-1 sexual transmission: early events of HIV-1 infection of human cervico-vaginal tissue in an optimized ex vivo model. Mucosal Immunol.

[20] Rodriguez-Garcia M, Fortier JM, Barr FD, Wira CR (2018). Aging impacts CD103(+) CD8(+) T cell presence and induction by dendritic cells in the genital tract. Aging Cell.

[21] Iijima N, Iwasaki A (2014). A local macrophage chemokine network sustains protective tissue-resident memory CD4 T cells. Science.

[22] Labuda JC, Pham OH, Depew CE, Fong KD, Lee BS, Rixon JA, McSorley SJ (2021). Circulating immunity protects the female reproductive tract from Chlamydia infection. Proc Natl Acad Sci U S A.

[23] Nguyen NDNT, Olsen AW, Lorenzen E, Andersen P, Hvid M, Follmann F, Dietrich J (2020) Parenteral vaccination protects against transcervical infection with Chlamydia trachomatis and generate tissue-resident T cells post-challenge. npj Vaccines 5:1–12. 10.1038/s41541-020-0157-x 10.1038/s41541-020-0157-x PMC6978417 31993218

[24] Tan HX, Wheatley AK, Esterbauer R, Jegaskanda S, Glass JJ, Masopust D, De Rose R, Kent SJ (2018). Induction of vaginal-resident HIV-specific CD8 T cells with mucosal prime-boost immunization. Mucosal Immunol.

[25] Shin H, Iwasaki A (2012). A vaccine strategy that protects against genital herpes by establishing local memory T cells. Nature.

[26] Gopinath S, Kim MV, Rakib T, Wong PW, van Zandt M, Barry NA, Kaisho T, Goodman AL, Iwasaki A (2018). Topical application of aminoglycoside antibiotics enhances host resistance to viral infections in a microbiota-independent manner. Nat Microbiol.

[27] Gopinath S, Lu P, Iwasaki A (2020). Cutting Edge: The Use of Topical Aminoglycosides as an Effective Pull in "Prime and Pull" Vaccine Strategy. J Immunol.

[28] Bagri P, Ghasemi R, McGrath JJC, Thayaparan D, Yu E, Brooks AG, Stämpfli MR, Kaushic C (2020). Estradiol Enhances Antiviral CD4(+) Tissue-Resident Memory T Cell Responses following Mucosal Herpes Simplex Virus 2 Vaccination through an IL-17-Mediated Pathway. J Virol.

[29] Davé VA, Cardozo-Ojeda EF, Mair F, Erickson J, Woodward-Davis AS, Koehne A, Soerens A, Czartoski J, Teague C, Potchen N, Oberle S, Zehn D, Schiffer JT, Lund JM, Prlic M (2021) Cervicovaginal Tissue Residence Confers a Distinct Differentiation Program upon Memory CD8 T Cells. 206:2937–48. 10.4049/jimmunol.2100166 %J The Journal of Immunology 10.4049/jimmunol.2100166 PMC8642491 34088770

[30] Zheng D, Liwinski T, Elinav E (2020). Interaction between microbiota and immunity in health and disease. Cell Res.

[31] Vitale SG, Ferrari F, Ciebiera M, Zgliczyńska M, Rapisarda AMC, Vecchio GM, Pino A, Angelico G, Knafel A, Riemma G, De Franciscis P, Cianci S (2021) The Role of Genital Tract Microbiome in Fertility: A Systematic Review. Int J Mol Sci 23. 10.3390/ijms23010180 10.3390/ijms23010180 PMC8745627 35008605

[32] Shahid M, Quinlivan JA, Peek M, Castaño-Rodríguez N, Mendz GL (2022). Is there an association between the vaginal microbiome and first trimester miscarriage? A prospective observational study. J Obstet Gynaecol Res.

[33] Arena B, Daccò MD (2021). Evaluation of vaginal microbiota in women admitted to the hospital for premature labour. Acta Biomed.

[34] Di Simone N, Santamaria Ortiz A, Specchia M, Tersigni C, Villa P, Gasbarrini A, Scambia G, D'Ippolito S (2020). Recent Insights on the Maternal Microbiota: Impact on Pregnancy Outcomes. Front Immunol.

[35] Bayar E, Bennett PR, Chan D, Sykes L, MacIntyre DA (2020). The pregnancy microbiome and preterm birth. Semin Immunopathol.

[36] Koren O, Goodrich JK, Cullender TC, Spor A, Laitinen K, Bäckhed HK, Gonzalez A, Werner JJ, Angenent LT, Knight R, Bäckhed F, Isolauri E, Salminen S, Ley RE (2012). Host remodeling of the gut microbiome and metabolic changes during pregnancy. Cell.

[37] Lee SK, Kim CJ, Kim DJ, Kang JH (2015). Immune cells in the female reproductive tract. Immune Netw.

[38] Gibbs A, Leeansyah E, Introini A, Paquin-Proulx D, Hasselrot K, Andersson E, Broliden K, Sandberg JK, Tjernlund A (2017). MAIT cells reside in the female genital mucosa and are biased towards IL-17 and IL-22 production in response to bacterial stimulation. Mucosal Immunol.

[39] Geppert M, Geppert J (1982). Lymphocytes in the epithelial layers of decidua and normal or abnormal endometrium. Arch Gynecol.

[40] Vallve-Juanico J, Houshdaran S, Giudice LC (2019). The endometrial immune environment of women with endometriosis. Hum Reprod Update.

[41] Yeaman GR, Collins JR, Fanger MW, Wira CR (2001) CD8+ T cells in human uterine endometrial lymphoid aggregates: evidence for accumulation of cells by trafficking. Immunology 102:434–440. 10.1046/j.1365-2567.2001.01199.x 10.1046/j.1365-2567.2001.01199.x PMC1783206 11328377

[42] Southcombe JH, Mounce G, McGee K, Elghajiji A, Brosens J, Quenby S, Child T, Granne I (2017). An altered endometrial CD8 tissue resident memory T cell population in recurrent miscarriage. Sci Rep.

[43] Filipovic I, Chiossone L, Vacca P, Hamilton RS, Ingegnere T, Doisne JM, Hawkes DA, Mingari MC, Sharkey AM, Moretta L, Colucci F (2018). Molecular definition of group 1 innate lymphoid cells in the mouse uterus. Nat Commun.

[44] Gamliel M, Goldman-Wohl D, Isaacson B, Gur C, Stein N, Yamin R, Berger M, Grunewald M, Keshet E, Rais Y, Bornstein C, David E, Jelinski A, Eisenberg I, Greenfield C, Ben-David A, Imbar T, Gilad R, Haimov-Kochman R, Mankuta D, Elami-Suzin M, Amit I, Hanna JH, Yagel S, Mandelboim O (2018). Trained Memory of Human Uterine NK Cells Enhances Their Function in Subsequent Pregnancies. Immunity.

[45] Givan AL, White HD, Stern JE, Colby E, Gosselin EJ, Guyre PM, Wira CR (1997). Flow cytometric analysis of leukocytes in the human female reproductive tract: comparison of fallopian tube, uterus, cervix and vagina. Am J Reprod Immunol.

[46] Glasser SR, Aplin JD, Giudice LC, Tabibzadeh S (2002) The Edometrium.

[47] Southcombe JH, Mounce G, McGee K, Elghajiji A, Brosens J, Quenby S, Child T, Granne I (2017). An altered endometrial CD8 tissue resident memory T cell population in recurrent miscarriage. Sci Rep.

[48] Moylan DC, Goepfert PA, Kempf MC, Saag MS, Richter HE, Mestecky J, Sabbaj S (2016). Diminished CD103 (alphaEbeta7) Expression on Resident T Cells from the Female Genital Tract of HIV-Positive Women. Pathog Immun.

[49] Woodward Davis AS, Vick SC, Pattacini L, Voillet V, Hughes SM, Lentz GM, Kirby AC, Fialkow MF, Gottardo R, Hladik F, Lund JM, Prlic M (2021). The human memory T cell compartment changes across tissues of the female reproductive tract. Mucosal Immunol.

[50] Wira CR, Fahey JV, Rodriguez-Garcia M, Shen Z, Patel MV (2014). Regulation of mucosal immunity in the female reproductive tract: the role of sex hormones in immune protection against sexually transmitted pathogens. Am J Reprod Immunol.

[51] Rodriguez-Garcia M, Shen Z, Fortier JM, Wira CR (2020). Differential Cytotoxic Function of Resident and Non-resident CD8+ T Cells in the Human Female Reproductive Tract Before and After Menopause. Front Immunol.

[52] Doisne JM, Balmas E, Boulenouar S, Gaynor LM, Kieckbusch J, Gardner L, Hawkes DA, Barbara CF, Sharkey AM, Brady HJ, Brosens JJ, Moffett A, Colucci F (2015). Composition, Development, and Function of Uterine Innate Lymphoid Cells. J Immunol.

[53] Matarese G, De Placido G, Nikas Y, Alviggi C (2003). Pathogenesis of endometriosis: natural immunity dysfunction or autoimmune disease?. Trends Mol Med.

[54] Giudice LC (2010). Clinical practice. Endometriosis N Engl J Med.

[55] Sojka DK, Plougastel-Douglas B, Yang L, Pak-Wittel MA, Artyomov MN, Ivanova Y, Zhong C, Chase JM, Rothman PB, Yu J, Riley JK, Zhu J, Tian Z, Yokoyama WM (2014). Tissue-resident natural killer (NK) cells are cell lineages distinct from thymic and conventional splenic NK cells. Elife.

[56] Giuliani E, Parkin KL, Lessey BA, Young SL, Fazleabas AT (2014). Characterization of uterine NK cells in women with infertility or recurrent pregnancy loss and associated endometriosis. Am J Reprod Immunol.

[57] Tanaka Y, Mori T, Ito F, Koshiba A, Takaoka O, Kataoka H, Maeda E, Okimura H, Mori T, Kitawaki J (2017). Exacerbation of Endometriosis Due To Regulatory T-Cell Dysfunction. J Clin Endocrinol Metab.

[58] Le NXH, Loret de Mola JR, Bremer P, Groesch K, Wilson T, Diaz-Sylvester P, Braundmeier-Fleming AG (2021). Alteration of systemic and uterine endometrial immune populations in patients with endometriosis. Am J Reprod Immunol.

[59] Bulletti C, Coccia ME, Battistoni S, Borini A (2010). Endometriosis and infertility. J Assist Reprod Genet.

[60] Thiruchelvam U, Wingfield M, O'Farrelly C (2016). Increased uNK Progenitor Cells in Women With Endometriosis and Infertility are Associated With Low Levels of Endometrial Stem Cell Factor. Am J Reprod Immunol.

[61] Glover LE, Crosby D, Thiruchelvam U, Harmon C, Chorcora CN, Wingfield MB, O'Farrelly C (2018). Uterine natural killer cell progenitor populations predict successful implantation in women with endometriosis-associated infertility. Am J Reprod Immunol.

[62] Durgeau A, Virk Y, Corgnac S, Mami-Chouaib F (2018). Recent Advances in Targeting CD8 T-Cell Immunity for More Effective Cancer Immunotherapy. Front Immunol.

[63] Monin L, Ushakov DS, Arnesen H, Bah N, Jandke A, Munoz-Ruiz M, Carvalho J, Joseph S, Almeida BC, Green MJ, Nye E, Hatano S, Yoshikai Y, Curtis M, Carlsen H, Steinhoff U, Boysen P, Hayday A (2020). gammadelta T cells compose a developmentally regulated intrauterine population and protect against vaginal candidiasis. Mucosal Immunol.

[64] Pinget GV, Corpuz TM, Stolp J, Lousberg EL, Diener KR, Robertson SA, Sprent J, Webster KE (2016). The majority of murine gammadelta T cells at the maternal-fetal interface in pregnancy produce IL-17. Immunol Cell Biol.

[65] Kabelitz D, Kalyan S, Oberg HH, Wesch D (2013). Human Vδ2 versus non-Vδ2 γδ T cells in antitumor immunity. Oncoimmunology.

[66] Peng G, Wang HY, Peng W, Kiniwa Y, Seo KH, Wang RF (2007). Tumor-infiltrating gammadelta T cells suppress T and dendritic cell function via mechanisms controlled by a unique toll-like receptor signaling pathway. Immunity.

[67] Webb JR, Milne K, Nelson BH (2014). Location, location, location: CD103 demarcates intraepithelial, prognostically favorable CD8(+) tumor-infiltrating lymphocytes in ovarian cancer. Oncoimmunology.

[68] Webb JR, Wick DA, Nielsen JS, Tran E, Milne K, McMurtrie E, Nelson BH (2010). Profound elevation of CD8+ T cells expressing the intraepithelial lymphocyte marker CD103 (alphaE/beta7 Integrin) in high-grade serous ovarian cancer. Gynecol Oncol.

[69] Komdeur FL, Prins TM, van de Wall S, Plat A, Wisman GBA, Hollema H, Daemen T, Church DN, de Bruyn M, Nijman HW (2017). CD103+ tumor-infiltrating lymphocytes are tumor-reactive intraepithelial CD8+ T cells associated with prognostic benefit and therapy response in cervical cancer. Oncoimmunology.

[70] Workel HH, Komdeur FL, Wouters MC, Plat A, Klip HG, Eggink FA, Wisman GB, Arts HJ, Oonk MH, Mourits MJ, Yigit R, Versluis M, Duiker EW, Hollema H, de Bruyn M, Nijman HW (2016). CD103 defines intraepithelial CD8+ PD1+ tumour-infiltrating lymphocytes of prognostic significance in endometrial adenocarcinoma. Eur J Cancer.

[71] Bosmuller HC, Wagner P, Peper JK, Schuster H, Pham DL, Greif K, Beschorner C, Rammensee HG, Stevanovic S, Fend F, Staebler A (2016). Combined Immunoscore of CD103 and CD3 Identifies Long-Term Survivors in High-Grade Serous Ovarian Cancer. Int J Gynecol Cancer.

[72] Webb JR, Milne K, Nelson BH (2015). PD-1 and CD103 Are Widely Coexpressed on Prognostically Favorable Intraepithelial CD8 T Cells in Human Ovarian Cancer. Cancer Immunol Res.

[73] Webb JR, Milne K, Kroeger DR, Nelson BH (2016). PD-L1 expression is associated with tumor-infiltrating T cells and favorable prognosis in high-grade serous ovarian cancer. Gynecol Oncol.

[74] Komdeur FL, Wouters MCA, Workel HH, Tijans AM, Terwindt ALJ, Brunekreeft KL, Plat A, Klip HG, Eggink FA, Leffers N, Helfrich W, Samplonoius DF, Bremer E, Wisman GB, Daemen T, Duiker EW, Hollema H, Nijman HW, de Bruyn M (2016). CD103+ intraepithelial T cells in high-grade serous ovarian cancer are phenotypically diverse TCRαβ+ CD8αβ+ T cells that can be targeted for cancer immunotherapy. Oncotarget.

[75] Vogler M, Shanmugalingam S, Särchen V, Reindl LM, Grèze V, Buchinger L, Kühn M, Ullrich E (2021). Unleashing the power of NK cells in anticancer immunotherapy. J Mol Med.

[76] Wendel P, Reindl LM, Bexte T, Künnemeyer L, Särchen V, Albinger N, Mackensen A, Rettinger E, Bopp T, Ullrich E (2021). Arming Immune Cells for Battle: A Brief Journey through the Advancements of T and NK Cell Immunotherapy. Cancers.

[77] Bister J, Crona Guterstam Y, Strunz B, Dumitrescu B, Haij Bhattarai K, Ozenci V, Brannstrom M, Ivarsson MA, Gidlof S, Bjorkstrom NK (2021). Human endometrial MAIT cells are transiently tissue resident and respond to Neisseria gonorrhoeae. Mucosal Immunol.

[78] Wagner M, Yoshihara M, Douagi I, Damdimopoulos A, Panula S, Petropoulos S, Lu H, Pettersson K, Palm K, Katayama S, Hovatta O, Kere J, Lanner F, Damdimopoulou P (2020). Single-cell analysis of human ovarian cortex identifies distinct cell populations but no oogonial stem cells. Nat commun.

[79] Wu R, Fujii S, Ryan NK, Van der Hoek KH, Jasper MJ, Sini I, Robertson SA, Robker RL, Norman RJ (2007). Ovarian leukocyte distribution and cytokine/chemokine mRNA expression in follicular fluid cells in women with polycystic ovary syndrome. Hum Reprod.

[80] Ardighieri L, Lonardi S, Moratto D, Facchetti F, Shih I-M, Vermi W, Kurman RJ (2014). Characterization of the immune cell repertoire in the normal fallopian tube. International journal of gynecological pathology : official journal of the International Society of Gynecological Pathologists.

[81] PrabhuDas M, Bonney E, Caron K, Dey S (2015). Immune mechanisms at the maternal-fetal interface: perspectives and challenges. Nat Immunol.

[82] Apps R, Murphy SP, Fernando R, Gardner L, Ahad T, Moffett A (2009). Human leucocyte antigen (HLA) expression of primary trophoblast cells and placental cell lines, determined using single antigen beads to characterize allotype specificities of anti-HLA antibodies. Immunology.

[83] Yadi H, Burke S, Madeja Z, Hemberger M, Moffett A, Colucci F (2008). Unique receptor repertoire in mouse uterine NK cells. J Immunol.

[84] King A, Balendran N, Wooding P, Carter NP, Loke YW (1991). CD3- leukocytes present in the human uterus during early placentation: phenotypic and morphologic characterization of the CD56++ population. Dev Immunol.

[85] Manaster I, Mandelboim O (2010). The unique properties of uterine NK cells. Am J Reprod Immunol.

[86] Chiossone L, Vacca P, Orecchia P, Croxatto D, Damonte P, Astigiano S, Barbieri O, Bottino C, Moretta L, Mingari MC (2014). In vivo generation of decidual natural killer cells from resident hematopoietic progenitors. Haematologica.

[87] Yang X, Yang Y, Yuan Y, Liu L, Meng T (2020). The Roles of Uterine Natural Killer (NK) Cells and KIR/HLA-C Combination in the Development of Preeclampsia: A Systematic Review. Biomed Res Int.

[88] Scherjon S (2020). Do we need to consider human leucocyte antigen-C typing in infertility treatment?. Fertil Steril.

[89] Feyaerts D, van der Meer A, Joosten I, van der Molen RG (2019). Selective expansion and CMV-dependency in pregnancy trained human endometrial NK cells. Cell Mol Immunol.

[90] Fan DX, Duan J, Li MQ, Xu B, Li DJ, Jin LP (2011). The decidual gamma-delta T cells up-regulate the biological functions of trophoblasts via IL-10 secretion in early human pregnancy. Clin Immunol.

[91] Nörenberg J, Meggyes M, Jakso P, Miko E, Barakonyi A (2019). TIM-3 and TIM-1 Could Regulate Decidual γδTCR Bright T Cells during Murine Pregnancy. J Immunol Res.

[92] Fan DX, Zhou WJ, Jin LP, Li MQ, Xu XH, Xu CJ (2019). Trophoblast-Derived CXCL16 Decreased Granzyme B Production of Decidual gammadelta T Cells and Promoted Bcl-xL Expression of Trophoblasts. Reprod Sci.

[93] Cai D, Tang Y, Yao X (2019). Changes of gammadeltaT cell subtypes during pregnancy and their influences in spontaneous abortion. J Reprod Immunol.

[94] Terzieva A, Dimitrova V, Djerov L, Dimitrova P, Zapryanova S, Hristova I, Vangelov I, Dimova T (2019) Early Pregnancy Human Decidua is Enriched with Activated, Fully Differentiated and Pro-Inflammatory Gamma/Delta T Cells with Diverse TCR Repertoires. Int J Mol Sci 20. 10.3390/ijms20030687 10.3390/ijms20030687 PMC6387174 30764544

[95] Akoto C, Chan CYS, Ravi K, Zhang W, Vatish M, Norris SA, Hemelaar J (2020). γδ T cell frequencies are altered in HIV positive pregnant South African women and are associated with preterm birth. PLoS ONE.

[96] Solders M, Gorchs L, Tiblad E, Gidlöf S, Leeansyah E, Dias J, Sandberg JK, Magalhaes I, Lundell AC, Kaipe H (2019). Recruitment of MAIT Cells to the Intervillous Space of the Placenta by Placenta-Derived Chemokines. Front Immunol.

[97] Kaipe H, Raffetseder J, Ernerudh J, Solders M, Tiblad E (2020). MAIT Cells at the Fetal-Maternal Interface During Pregnancy. Front Immunol.

[98] Meggyes M, Szanto J, Lajko A, Farkas B, Varnagy A, Tamas P, Hantosi E, Miko E, Szereday L (2018) The possible role of CD8+/Vα7.2+/CD161++ T (MAIT) and CD8+/Vα7.2+/CD161(lo) T (MAIT-like) cells in the pathogenesis of early-onset pre-eclampsia. Am J Reprod Immunol 79. 10.1111/aji.12805 10.1111/aji.12805 29265516

[99] Boyson JE, Rybalov B, Koopman LA, Exley M, Balk SP, Racke FK, Schatz F, Masch R, WIlson SB, Strominger JL,  (2002). CD1d and invariant NKT cells at the human maternal–fetal interface. Proc Natl Acad Sci U S A.

[100] Matsumoto J, Kawana K, Nagamatsu T, Schust DJ, Fujii T, Sato H, Hyodo H, Yasugi T, Kozuma S, Taketani Y (2008). Expression of surface CD1d in the extravillous trophoblast cells of early gestational placenta is downregulated in a manner dependent on trophoblast differentiation. Biochem Biophys Res Commun.

[101] Boyson JE, Aktan I, Barkhuff DA, Chant A (2008). NKT cells at the maternal-fetal interface. Immunol Invest.

[102] Mostrom MJ, Scheef EA, Sprehe LM, Szeltner D, Tran D, Hennebold JD, Roberts VHJ, Maness NJ, Fahlberg M, Kaur A (2021). Immune Profile of the Normal Maternal-Fetal Interface in Rhesus Macaques and Its Alteration Following Zika Virus Infection. Front Immunol.

[103] Boyson JE, Nagarkatti N, Nizam L, Exley M, Strominger JL (2006). Gestation stage-dependent mechanisms of invariant natural killer T cell-mediated pregnancy loss. Proc Natl Acad Sci U S A.

[104] Hoya M, Nagamatsu T, Fujii T, Schust DJ, Oda H, Akiba N, Iriyama T, Kawana K, Osuga Y, Fujii T (2018) Impact of Th1/Th2 cytokine polarity induced by invariant NKT cells on the incidence of pregnancy loss in mice. Am J Reprod Immunol 79. 10.1111/aji.12813 10.1111/aji.12813 29363849

[105] Li LP, Fang YC, Dong GF, Lin Y, Saito S (2012). Depletion of invariant NKT cells reduces inflammation-induced preterm delivery in mice. J Immunol.

[106] Li L, Yang J, Jiang Y, Tu J, Schust DJ (2015). Activation of decidual invariant natural killer T cells promotes lipopolysaccharide-induced preterm birth. Mol Hum Reprod.

[107] Hashemi V, Dolati S, Hosseini A, Gharibi T, Danaii S, Yousefi M (2017). Natural killer T cells in Preeclampsia: An updated review. Biomed Pharmacother.

[108] Tang L-C, Xu X-H, Jin L-P (2020). Molecular characteristics and possible functions of innate lymphoid cells in the uterus and gut. Cytokine Growth Factor Rev.

[109] Vacca P, Montaldo E, Croxatto D, Loiacono F, Canegallo F, Venturini PL, Moretta L, Mingari MC (2015). Identification of diverse innate lymphoid cells in human decidua. Mucosal Immunol.

[110] Mariotti FR, Quatrini L, Munari E, Vacca P, Moretta L (2019) Innate Lymphoid Cells: Expression of PD-1 and Other Checkpoints in Normal and Pathological Conditions. 10. 10.3389/fimmu.2019.00910 10.3389/fimmu.2019.00910 PMC6498986 31105707

[111] Xu Y, Romero R, Miller D, Silva P, Panaitescu B, Theis KR, Arif A, Hassan SS, Gomez-Lopez N (2018). Innate lymphoid cells at the human maternal-fetal interface in spontaneous preterm labor. Am J Reprod Immunol.

[112] Walker JA, McKenzie ANJ (2013). Development and function of group 2 innate lymphoid cells. Curr Opin Immunol.

[113] Bird L (2016). ILC2s drive allergen recall. Nat Rev Immunol.

[114] Liu L, Huang X, Xu C, Chen C, Zhao W, Li D, Li L, Wang L, Du M (2020). Decidual CD8(+)T cells exhibit both residency and tolerance signatures modulated by decidual stromal cells. J Transl Med.

[115] Huang X, Liu L, Xu C, Peng X, Li D, Wang L, Du M (2020). Tissue-resident CD8(+) T memory cells with unique properties are present in human decidua during early pregnancy. Am J Reprod Immunol.

[116] Streilein JW, Wegmann TG (1987). Immunologic privilege in the eye and the fetus. Immunol Today.

[117] Kahn DA, Baltimore D (2010). Pregnancy induces a fetal antigen-specific maternal T regulatory cell response that contributes to tolerance. Proc Natl Acad Sci U S A.

[118] Thiele K, Ahrendt LS, Hecher K, Arck PC (2019). The mnemonic code of pregnancy: Comparative analyses of pregnancy success and complication risk in first and second human pregnancies. J Reprod Immunol.

[119] Patel DA, Burnett NM, Curtis KM (2003) Reproductive Tract Infections. U.S. Department of Health and Human Services, U.S.A

[120] Pudney J, Anderson D (2011). Innate and acquired immunity in the human penile urethra. J Reprod Immunol.

[121] Fijak M, Meinhardt A (2006). The testis in immune privilege. Immunol Rev.

[122] Kaur G, Wright K, Verma S, Haynes A, Dufour JM, Cheng CY, Sun F (2021). The Good, the Bad and the Ugly of Testicular Immune Regulation: A Delicate Balance Between Immune Function and Immune Privilege. Molecular Mechanisms in Spermatogenesis, edn.

[123] Duan YG, Chen S, Haidl G, Allam JP (2017) Detection of invariant natural killer T cells in ejaculates from infertile patients with chronic inflammation of genital tract. Am J Reprod Immunol 78. 10.1111/aji.12671 10.1111/aji.12671 28371089

[124] De Rose R, Fernandez CS, Hedger MP, Kent SJ, Winnall WR (2013). Characterisation of macaque testicular leucocyte populations and T-lymphocyte immunity. J Reprod Immunol.

[125] Mukasa A, Hiromatsu K, Matsuzak G, O'Brien RL, Born W, Nomoto K (1995). Bacterial Infection of the Testis Leading to Autoaggressive Immunity Triggers Apparently Opposed Responses of αβ and γδT Cells. J Immunol.

[126] Tompkins AB, Hutchinson P, de Kretser DM, Hedger MP (1998). Characterization of lymphocytes in the adult rat testis by flow cytometry: effects of activin and transforming growth factor beta on lymphocyte subsets in vitro. Biol Reprod.

[127] Shamekh R, El-Badri NS, Saporta S, Pascual C, Sanberg PR, Cameron DF (2006). Sertoli Cells Induce Systemic Donor-Specific Tolerance in Xenogenic Transplantation Model. Cell Transplant.

[128] Tung KS, Harakal J, Qiao H, Rival C, Li JC, Paul AG, Wheeler K, Pramoonjago P, Grafer CM, Sun W, Sampson RD, Wong EW, Reddi PP, Deshmukh US, Hardy DM, Tang H, Cheng CY, Goldberg E (2017). Egress of sperm autoantigen from seminiferous tubules maintains systemic tolerance. J Clin Invest.

[129] Silva CA, Cocuzza M, Carvalho JF, Bonfá E (2014). Diagnosis and classification of autoimmune orchitis. Autoimmun Rev.

[130] Munoz G, Posnett DN, Witkin SS (1992). Enrichment of γδ T lymphocytes in human semen: relation between γδ T cell concentration and antisperm antibody status. J Reprod Immunol.

[131] Wilharm A, Brigas HC, Sandrock I, Ribeiro M, Amado T, Reinhardt A, Demera A, Hoenicke L, Strowig T, Carvalho T, Prinz I, Ribot JC (2021). Microbiota-dependent expansion of testicular IL-17-producing Vγ6+ γδT cells upon puberty promotes local tissue immune surveillance. Mucosal Immunol.

[132] Mukasa A, Lahn M, Pflum EK, Born W, O'Brien RL (1997). Evidence that the same gamma delta T cells respond during infection-induced and autoimmune inflammation. J Immunol.

[133] Ferlay J, Soerjomataram I, Dikshit R, Eser S, Mathers C, Rebelo M, Parkin DM, Forman D, Bray F (2015). Cancer incidence and mortality worldwide: sources, methods and major patterns in GLOBOCAN 2012. Int J Cancer.

[134] Kopp HG, Kuczyk M, Classen J, Stenzl A, Kanz L, Mayer F, Bamberg M, Hartmann JT (2006). Advances in the treatment of testicular cancer. Drugs.

[135] Ritzén EM (1990). Testicular relapse of acute lymphoblastic leukemia (ALL). J Reprod Immunol.

[136] Schenkel JM, Fraser KA, Vezys V, Masopust D (2013). Sensing and alarm function of resident memory CD8(+) T cells. Nat Immunol.

[137] Schenkel JM, Fraser KA, Beura LK, Pauken KE, Vezys V, Masopust D (2014). Resident memory CD8 T cells trigger protective innate and adaptive immune responses. Science.

[138] Burzyn D, Benoist C, Mathis D (2013). Regulatory T cells in nonlymphoid tissues. Nat Immunol.

[139] Hayday AC (2009). γδ T Cells and the Lymphoid Stress-Surveillance Response. Immunity.

[140] Chaplin DD (2010). Overview of the immune response. J Allergy Clin Immunol.

[141] Heilig JS, Tonegawa S (1986). Diversity of murine gamma genes and expression in fetal and adult T lymphocytes. Nature.

[142] Meermeier EW, Harriff MJ, Karamooz E, Lewinsohn DM (2018). MAIT cells and microbial immunity. Immunol Cell Biol.

[143] Gibbs A, Leeansyah E, Introini A, Paquin-Proulx D, Hasselrot K, Andersson E, Broliden K, Sandberg JK, Tjernlund A (2017). MAIT cells reside in the female genital mucosa and are biased towards IL-17 and IL-22 production in response to bacterial stimulation. Mucosal Immunol.

[144] Lee M, Lee E, Han SK, Choi YH, Kwon D-i, Choi H, Lee K, Park ES, Rha M-S, Joo DJ, Shin E-C, Kim S, Kim JK, Lee YJ (2020). Single-cell RNA sequencing identifies shared differentiation paths of mouse thymic innate T cells. Nat Commun.

[145] Kronenberg M (2005). Toward an understanding of NKT cell biology: progress and paradoxes. Annu Rev Immunol.

[146] Grégoire C, Chasson L, Luci C, Tomasello E, Geissmann F, Vivier E, Walzer T (2007). The trafficking of natural killer cells. Immunol Rev.

[147] Gronke K, Kofoed-Nielsen M, Diefenbach A (2016). Innate lymphoid cells, precursors and plasticity. Immunol Lett.

[148] Tang LC, Xu XH, Jin LP (2020). Molecular characteristics and possible functions of innate lymphoid cells in the uterus and gut. Cytokine Growth Factor Rev.

[149] Vacca P, Vitale C, Munari E, Cassatella MA, Mingari MC, Moretta L (2018). Human Innate Lymphoid Cells: Their Functional and Cellular Interactions in Decidua. Front Immunol.

[150] Hao F, Zhou X, Jin L (2020). Natural killer cells: functional differences in recurrent spontaneous abortion†. Biol Reprod.

[151] Boulenouar S, Doisne JM, Sferruzzi-Perri A, Gaynor LM, Kieckbusch J, Balmas E, Yung HW, Javadzadeh S, Volmer L, Hawkes DA, Phillips K, Brady HJ, Fowden AL, Burton GJ, Moffett A, Colucci F (2016). The Residual Innate Lymphoid Cells in NFIL3-Deficient Mice Support Suboptimal Maternal Adaptations to Pregnancy. Front Immunol.

